# Oxytocin administration for induction and augmentation of labour in polish maternity units – an observational study

**DOI:** 10.1186/s12884-021-04190-w

**Published:** 2021-11-11

**Authors:** Barbara Baranowska, Anna Kajdy, Iwona Kiersnowska, Dorota Sys, Urszula Tataj-Puzyna, Déirdre Daly, Michał Rabijewski, Grażyna Bączek, Maria Węgrzynowska

**Affiliations:** 1 Department of Midwifery, Centre of Postgraduate Medical Education, Warsaw, Poland; 2 Department of Reproductive Health, Centre of Postgraduate Medical Education, 90 Żelazna St., 01-004 Warsaw, Poland; 3 Department of Obstetrics and Perinatology, Medical University of Warsaw, Warsaw, Poland; 4 School of Nursing and Midwifery, Trinity College Dublin, Dublin, Ireland; 5 Department of Gynecologic and Obstetrical Didactics, Medical University of Warsaw, Warsaw, Poland

**Keywords:** Oxytocin, Labour, Induction, Augmentation, Practice protocol

## Abstract

**Background:**

There is not enough data regarding practices and protocols on the dose of oxytocin administrated to women during labour. Empirical evidence indicates that compliance with the guidelines improves the quality of healthcare and reduces adverse effects. The study aimed to evaluate practices of oxytocin provision for labour induction and augmentation in two maternity units in Poland.

**Methods:**

The article presents a prospective observational study. Data from 545 (*n* = 545) labours was collected in two maternity units. First, the total dose (the total amount of oxytocin provided from the beginning in the labour ward until delivery including the III and IV stage of labour) and cumulative dose of oxytocin (the amount of oxytocin given until the birth of the neonate) administered to women during labour was calculated. Then, the relationship between the cumulative dose of oxytocin and short term perinatal outcomes (mode of delivery, use of epidural anaesthesia, Apgar scores, birth weight and postpartum blood loss) was analysed. Finally, the compliance of oxytocin supply during labour with national guidelines in the following five criteria: medium, start dose, escalation rate, interval, the continuation of infusion after established labour was examined.

**Results:**

The average cumulative dose of oxytocin administrated to women before birth was 4402 mU following labour induction and 2366 mU following labour augmentation. The actual administration of oxytocin deviated both from the unit and national guidelines in 93.6% of all observed labours (mainly because of continuation of infusion after established labour). We found no statistically significant correlation between the cumulative dose of oxytocin administered and mode of delivery, immediate postpartum blood loss or Apgar scores. There was no observed effect of cumulative dose oxytocin on short-term perinatal outcomes. The two units participating in the study had similar protocols and did not differ significantly in terms of total oxytocin dose, rates of induction and augmentation - the only observed difference was the mode of delivery.

**Conclusions:**

The study showed no effect of the mean cumulative oxytocin dose on short-term perinatal outcomes and high rate of non-compliance of the practice of oxytocin administration for labour induction and augmentation with the national recommendations. Cooperation between different professional groups of maternity care providers should be considered in building national guidelines for maternity care.. Further studies investigating possible long-term effects of the meant cumulative dose of oxytocin and the reasons for non-compliance of practice with guidelines should be carried out.

**Supplementary Information:**

The online version contains supplementary material available at 10.1186/s12884-021-04190-w.

## Background

The number of women who have their labour induced or augmented with synthetic oxytocin is increasing in high-income countries throughout the world [1, 2]. At the same time, there are considerable differences in the intrapartum oxytocin administration regimens between and within countries, regions and hospitals in Europe [3]. In Poland, there is no official data regarding oxytocin administration. Surveys addressed to women who had given birth revealed that between 43 and 63% of all births are induced or stimulated with synthetic oxytocin [4]. In 2017, the Polish Society of Gynaecologists and Obstetricians (PSOGO https://www.ptgin.pl/) issued national guidelines on the use of synthetic oxytocin for labour induction and augmentation [5]. They recommend two oxytocin administration regimens: (i) low dose (start dose 0.5–2 milliUnits per minute (mU/min), escalating at 1–2 mU/min at 15–40 min intervals) and (ii) high dose (start dose 6 mU/min, escalating at 3-6 mU/min at 15–40 min intervals). The guidelines also state that there are no apparent benefits to continuing oxytocin infusion after achieving effective uterine contractions and reaching the active phase of labour (dilatation > 5 cm). The guidelines on the use of synthetic oxytocin for labour induction and augmentation do not include case-specific variations (obese women, multiple pregnancies etc).

However, there is not enough data regarding practices and protocols that healthcare personnel follow and the dose of oxytocin that women receive during labour. Observed differences in the practice of oxytocin use should stimulate efforts to standardise the procedures [3]. Following a strict protocol may face resistance from those responsible for labour care [6].

Routine oxytocin use presents no benefits when there are no medical indications for labour augmentation [7, 8]. Despite its widespread use during labour, and its effectiveness in labour induction and augmentation [9], there is still little research on the short and long-term consequences of synthetic oxytocin on both the woman and child [10].

While an increasing number of studies suggest possible adverse effects of intrapartum synthetic oxytocin [11], there is little research on the impact on the mother and the baby of the total and cumulative dose of synthetic oxytocin administered during labour [12–15]. These studies concentrate primarily on the effects of maternal obesity and indicate that significantly higher dosages of oxytocin are used in obese women. Additionally, the Carlson study shows that hourly oxytocin dose in obese women was also related to neonatal birth weight and cervical dilation at oxytocin initiation [12].

Studying these aspects is particularly crucial in the light of the latest reports on the longer than the previously believed half-life of oxytocin that can lead to high accumulation and unnecessary exposure in women [16].

This study aimed to assess administration practices of oxytocin for labour induction and augmentation in two maternity units in Poland. First, the assessment included calculation of the total amount of oxytocin provided from the beginning of labour until delivery (including the III and IV stage of labour) and cumulative dose of oxytocin administered to women during labour (the amount of oxytocin given until the birth of the neonate). Then, the relationship between the cumulative dose of oxytocin and short term perinatal outcomes (mode of delivery, use of epidural anaesthesia, Apgar scores, birth weight and postpartum blood loss) was analysed. Finally, compliance of administration practices with national guidelines was analysed.

## Methods

### Study design and setting

The study was conducted between January 1 and July 312,019, in Warsaw, Poland. Currently, there are 378 maternity units in Poland, organised on a three-level referral system, with tertiary hospitals providing the most specialist care [17]. Sixteen of these maternity units are in Warsaw, catering for approximately 21,000 births per annum [18]. In 2018 in Poland, the caesarean section (CS) rate was 43.9% (including elective and emergency CS), and labour induction and augmentation rates were 43 and 61%, respectively [4, 19]. Poland has one of the highest rates of caesarean section among 21 OECD countries (the Organization for Economic Co-operation and Development) [20].

### Data collection

Sixteen maternity units were invited to participate in the study. The inclusion criteria for maternity units was consent to participate and implemented internal written protocols compatible with the national guidelines for oxytocin administration during labour. Five hospitals did not consent to participate in the study. Eight hospitals did not have a written protocol. One hospital had a written protocol, but it did not adhere to the national guidelines. Two hospitals fulfilled the established inclusion criteria (unit A and B). Both of these units were tertiary hospitals. In those units, contemporaneous observation of labour assessing oxytocin use was performed by trained volunteer midwives between January 15 and July 312,019. Midwives working in labour and delivery wards in both units were invited to volunteer their participation in the study. Volunteer midwives were trained on how to record intrapartum oxytocin administration and complete the data collection form [see Additional file 1]. Convenience sampling was used to collect the data. Trained midwives observed and filled the data collection forms of induced or augmented labours during their planned shifts. The inclusion criteria were women in term pregnancies, women > 18 years of age, no known foetal abnormalities.

Data were collected on maternal age, parity, gestational age, indication(s) for induction or augmentation of labour, type and volume of infusion solution (ml), dose of oxytocin in the infusion (International Units - IU), start dose (mU/min), maximum dose (mU/min), escalation rate (mU/min) and exact time of each escalation (interval) (min), use of epidural anaesthesia, Apgar scores and blood loss (ml). The total dose of oxytocin administrated during labour is defined as the total amount of oxytocin provided from the beginning of labour until delivery (including the III and IV stage of labour) and calculated in milliUnits (mU). The cumulative dose is defined as the amount of oxytocin given until the birth of the neonate (excluding the IV stage of labour) and is calculated in milliUnits (mU). Short term perinatal outcomes assessed in the study were the mode of delivery, use of epidural anaesthesia, Apgar scores, birth weight and blood loss.

In cases of emergency CS, we excluded the dose of oxytocin administered after birth. The total time of oxytocin administration was calculated from the start of the infusion until delivery. The time during which the infusion was stopped/disconnected (e.g., for the administration of epidural anaesthesia) was deducted from the administration time. The protocol in unit A included: Infusion medium - 5 IU oxytocin in 0.9% NaCl 50 ml, Starting dose – 1.67 mU/min, Maximum dose - 10 mU/min, Escalation rate – 1.67 mU/min every 10–15 min. In unit B: Infusion fluid - 5 IU oxytocin in Glucose 50 ml, Starting dose - 1-2 mU/min, Maximum dose - 20 mU/min, Escalation rate - 1-2 mU/min every 30 mins. None of the protocols stated contraindications and case specific variations (for example multiple pregnancy and obesity).

We assessed each observation for compliance with the national protocols in all the defined criteria: medium, start dose, escalation rate, interval, the continuation of infusion after established labour. We achieved the sample size through convenience sampling over a limited time, as described above (244 births in unit A and 301 in unit B). There was no prespecified sample size calculated because no comparisons regarding outcome and interventions were made between the studied units.

### Data analysis

All analysis was conducted using statistical program R with statistical significance set at p = < 0.05 for all analysis [21]. Nominal variables were compared using the Chi-square test. Ordinal variables were checked for normality of distribution, using the Shapiro-Wilk test and compared with the ANOVA Kruskal-Wallis and U Mann-Whitney tests. Correlations between ordinal variables were analysed using Spearman test, and correlations between nominal variables were calculated using a linear model. The results are presented as an average and standard deviation and as numbers and percentages of the total. Correlations with induction or augmentation of labour and mode of birth were calculated using Wilcoxon rank-sum test with continuity correlation.

### Ethical issues

The Bio-ethical Commission approved the study of the Medical University of Warsaw (reference number AKB/226/2018). According to Polish law, non-interventional observational studies do not require patient consent, which was confirmed by the Bio-ethics Committee assessing this study. The consent of the Hospital Director was obtained for access to medical data.

## Results

The data on 545 births were analysed, 244 births in unit A and 301 in unit B (Supplementary Table 1). Women’s average age was 31 years [range 19 to 44 years, SD = 4.5], 68% (*n* = 375) were nulliparous. In our study the rate of augmentation of labour was 33% (*n* = 182), while the percentage of induction was 67% (*n* = 363). In the studied group, most deliveries were vaginal (84%; *n* = 456), and 16% (*n* = 89) ended up in a caesarean section. In unit A caesarean section rate was 13% and in Unit B 20%. The most common indications for labour induction were maternal gestational diabetes mellitus, pregnancy-induced hypertension, small for gestational age (foetal growth ≤10th percentile for gestational weight) and prelabour rupture of membranes. The only indication for labour augmentation was hypotonic uterine action (weakening of uterine contractions in the first and second stage of labour). The maternal characteristics and obstetric outcome from the two units are presented in Table 1.

**Table 1:** The maternal characteristics and obstetric outcome at the two study sites

	Unit A *N* = 244	Unit B *N* = 301	Total N = 545	p -value
	n (%)	n (%)	n (%)	
Age (years)				*p* = 0.117 χ2 = 4,29
≤24	16 (7)	20 (7)	36 (7)	
25–34	162 (66)	222 (73)	384 (70)	
≥ 35	66 (27)	59 (20)	125 (23)	
Gestational age (weeks)				*p* = 0.000* χ2 = 16,67
37–39	108 (44)	186 (62)	294 (54)	
> 40	136 (56)	115 (38)	251 (46)	
Parity				*p* = 0.576 χ2 = 4,81
Nulliparous	165 (68)	210 (70)	375 (69)	
Multiparous	79 (32)	91 (30)	170 (31)	
Type of delivery				*p* = 0.021* χ2 = 5,26
Vaginal delivery	214 (88)	242 (80)	456 (84)	
Cesarean sections	30 (12)	59 (20)	89 (16)	
Induction	161 (66)	202 (67)	363 (67)	*p* = 0.802 χ2 = 0,06
Augmentation	83 (34)	99 (33)	182 (33)	
Postpartum hemorrhage (ml)^a^				p = 0.000* χ2 = 127,08
< 300	8 (4)	123 (51)	131 (29)	
301–500	199(93)	108 (45)	307 (67)	
> 501	7 (3)	11 (4)	18 (4)	
Epidural				p = 0.000* χ2 = 38,65
Yes	92 (38)	194 (64)	286 (52)	
No	152 (62)	107 (36)	259 (48)	
Apgar score (points)				–
1 min < 7	1 (0.4)	1 (0.3)	2 (0.4)	
5 min < 7	2 (0.8)	0	2 (0.4)	
10 min < 7	0	0	0	
Birth weight (g)				*p* = 0.924 χ^2^ = 0,09
< 4000	224 (92)	277 (92)	501 (82)	
≥4000	20 (8)	24 (8)	44 (8)	

*p-value (*p* < 0,05)

^a^ only vaginal deliveries were included

In the studied group, after induction of labour (n = 363), 81% of women had vaginal deliveries (*n* = 295) (Table 2). Nulliparous had higher rates of caesarean sections than multiparas (25% v 6%, chi2 = 18,34; *p* < 0.05). In the group with augmented labour (*n* = 182), 88% delivered vaginally (*n* = 161) (Table 2). Among nulliparous and multiparas, the caesarean rate was 4, and 14% respectively, but there were no statistical differences between the groups.

**Table 2:** Maternal, obstetric and neonatal data in relation to the mean cumulative oxytocin dose, maximum oxytocin dose and interval in induction and augmentation groups

	Induction *N* = 363				Augmentation *N* = 182			
	n (%)	Cumulative dose (mU) (mean ± SD)	Maximum dose (mU/min) (mean ± SD)	Interval (min) (mean ± SD)	n (%)	Cumulative dose (mU) (mean ± SD)	Maximum dose (mU/min) (mean ± SD)	Interval (min) (mean ± SD)
Age (years)								
≤24	19 (5)	3276 (±2297)	15.49 (±8.84)	55 (±30)	17 (9)	2524 (±1490)	15.59 (±9.29)	48 (±41)
25–34	250 (69)	4702 (±3128)	19.42 (±8.97)	62 (±38)	134 (74)	2461 (±2086)	15.77 (±8.23)	34 (±22)
≥ 35	94 (26)	3859 (±4423)	17.45 (±9.85)	56 (±34)	31 (17)	1875 (±1649)	14.97 (±9.55)	30 (±25)
Gestational age (weeks)								
37–39	217 (60)	4519 (±3138)	19,29 (±9.40)	57 (±38)	77 (42)	2208 (±1778)	14.40 (±7.67)	33 (±21)
> 40	146 (40)	4246 (±3975)	17,84 (±8.98)	59 (±45)	105 (58)	2482 (±2106)	16.50 (±9,03)	35 (±25)
Parity								
nulliparous	243 (67)	5108 (±3237)	20,61 (±9.68)	65 (±42)	132 (73)	2704 (±2078)	16,85 (±8,37)	34 (±23)
multiparous	120 (33)	3017 (±3592)	14,91 (±9.95)	44 (±35)	50 (27)	1474 (±1314)	12,35(±8,09)	35 (±29)
Type of delivery								
Vaginal delivery	295 (81)	4267 (±3350)	18,96 (±9.21)	58 (±42)	161 (88)	2394 (±1941)	15,85 (±8,43)	34 (±21)
Cesarean sections	68 (19)	5010 (±4033)	17,61 (±9.43)	57 (±39)	21 (12)	2154 (±2247)	13,82 (±9.21)	34 (±25)
Postpartum hemorrhage (ml)^a^								
< 300	86 (29)	3686 (±2436)	13,36 (±7.07)	53 (±39)	45 (28)	1959 (±1605)	13,36 (±7.07)	29 (±20)
301–500	198 (67)	4483 (±3709)	16,74 (±8.84)	62 (±41)	109 (68)	2474 (±2014)	16,74 (±8.84)	37 (±27)
> 501	11 (4)	4891 (±2049)	17,00 (±7.07)	60 (±49)	7 (4)	3800 (±2070)	17,00 (±7.07)	40 (±21)
Epidural								
Yes	171 (47)	5132 (±4022)	20,27 (±9.64)	60 (±43)	114 (63)	2530 (±1890)	16,87 (±8.62)	33 (±23)
No	192 (53)	3754 (±2796)	17,29 (±8.67)	56 (±39)	68 (37)	2091 (±2090)	13,50 (±7.95)	35 (±27)
Birth weight (g)								
< 4000	337 (93)	4351 (±3532)	18,62 (±9.34)	56 (±38)	164 (90)	2284 (±1820)	15,26 (±8.34)	35 (±26)
≥4000	26 (7)	5188 (±2936)	19,85 (±7.96)	80 (±67)	18 (10)	3165 (±3059)	19,00 (±9.65)	29 (±13)

^a^ only vaginal deliveries were included

In both units, the cumulative dose of oxytocin administered was considerably higher when labour was induced (*p* < 0.05). Women that underwent induction of labour, on average received, 4402 mU (±3495) of oxytocin, while women during augmented labour received 2366 mU (±1973). The total dose of oxytocin administered until childbirth and after birth to women during labour was 7513 mU (±3813) for induction of labour and 9617 mU (±4586) for augmentation of labour, a statistically significant difference (*p* < 0.05).

Without exception, the oxytocin infusion was continued after 5 cm cervical dilatation following induction of labour and discontinued only after the baby’s birth.

The minimum escalation rate was 1.67 mU/min and maximum was 58.4 mU/min [median 9.3 mU/min, average 10.3 mU/min]. The minimum interval was 2 min, and a maximum of 480 min (median 43.3 min, average 54.1 min).

The cumulative dose of oxytocin related to maternal characteristics and different obstetric outcome is presented in Table 3.

**Table 3:** The cumulative dose oxytocin related to maternal characteristics and different obstetric outcome

	n (%)	cumulative dose oxytocin mU (mean ± SD)	*p*-value
Age (years)			*p* = 0.087
≤24	36 (7)	2921(±1968)	
25–34	384 (70)	3913 (±3000)	
≥ 35	125 (23)	3367 (±4009)	
Gestational age (weeks)			*p* = 0.016*
37–39	294 (54)	3912 (±3019)	
> 40	251 (46)	3503 (±3425)	
Parity			p = 0.000*
Nulliparous	375 (69)	4251 (±3098)	
Multiparous	170 (31)	2566 (±3178)	
Type of delivery			*p* = 0.358
Vaginal delivery	456 (84)	3599 (±3059)	
Cesarean sections	89 (16)	4344 (±3878)	
Oxytocin (indication)			p = 0.000*
Induction	363 (67)	4409(±3497)	
Augmentation	182 (33)	2366 (±1973)	
Postpartum blood loss (ml)			*p* = 0.472
< 300	131 (29)	3106 (±2334)	
301–500	307 (67)	3753 (±3338)	
> 501	18 (4)	4467 (±2069)	
Epidural			*p* = 0.007*
Yes	286 (53)	4088 (±3567)	
No	259 (47)	3314 (±2725)	
Birth weight			*p* = 0.122
< 4000	501 (92)	3611 (±3224)	
≥4000	44 (8)	4313 (±3099)	

*p-value (*p* < 0.05)

There was a negative correlation between the cumulative dose of oxytocin administered during labour and parity (R Spearman = − 0.4087, *p* < 0.05), and a weak correlation with duration of pregnancy (R Spearman = − 0.138, *p* < 0.05). There was no statistically significant correlation between the dose of oxytocin administered and maternal age (*p* = 0.2669).

With the increasing cumulative dose of oxytocin, the use of epidural anaesthesia increased slightly (Estimate = 0.04908, Pr(>IzI) = 0.005876).

There was no statistically significant correlation between the cumulative dose of oxytocin administered and the rate of emergency CS (*p* = 0.05926), forceps-assisted (*p* = 0.3884) or vacuum-assisted births (*p* = 0.7281), immediate postpartum blood loss (*p* = 0.7609), Apgar scores (*p* = 0.8908) and birth weight (*p* = 0.2015). Similarly, there were no statistically significant differences between the units in the total dose of oxytocin administrated to women.

In most instances (93.6%), the actual administration of oxytocin did not comply with the PSOGO guidelines mainly due to continuation of infusion after achieving effective uterine contractions and beginning of the active phase of labour (dilatation > 5 cm) (Table 4). In regard to the start dose, escalation rate and interval, the protocol was followed in 18.9% (*n* = 103) of labours: 18.4% (*n* = 100) followed the low-dose protocol (start dose 0.5–2 mU/min, escalation rate 1–2 mU/min, interval 15–40 min) and 0.6% (*n* = 3) followed the high-dose protocol (start dose 6 mU/min, escalation rate 3-6 mU/min, interval 15–40 min). The most common example of non-compliance in regards to escalation rates and intervals were higher than recommended escalation rates and longer intervals.

**Table 4:** Adherence of practice of oxytocin administration to the national guidelines (*N* = 545)

	VARIABLES IN ADHERANCE WITH NATIONAL PROTOCOL					
	Medium (*n* = 545)	Start dose^a^ (*n* = 509)	Escalation rate (*n* = 545)	Interval (*n* = 545)	Continuation of infusion after established labour was achieved^b^ (*n* = 363)	All variables of accordance present (*n* = 545)
Compliant	n = 545 (100%)	*n* = 130 (26%)	*n* = 131 (24%)	*n* = 192 (35%)	n = 4 (1%)	*n* = 35 (6%)
Non-compliant	*n* = 0 (0%)	*n* = 379 (74%)	*n* = 414 (76%)	*n* = 353 (65%)	*n* = 541 (99%)	*n* = 510 (94%)

^a^36 patients had previously administered oxytocin – start dose not available

^b^ only induced labours

## Discussion

In our study, the mean cumulative oxytocin dose was 3387 mU and mean total oxytocin dose was 8565 mU. The mean cumulative dose of oxytocin and the total dose of oxytocin administrated to women in our study following augmentation was similar to results in the study by Roloff et al. [14]. The total dose of oxytocin reported by Selin et al. (2019) was higher than the total dose found in our study [22]. Similar to our findings, Frey et al. (2015) found that induction was associated with higher maximum oxytocin doses [23]. The fact that higher doses of oxytocin were administrated to women following induction of labour is understandable, given that the infusion is likely to be continued for a more extended period.

Furthermore, there was a correlation between the mean cumulative dose of oxytocin infused, parity and duration of pregnancy. Similar correlations between parity were reported by Oscarssona et al. (2006). Multiparous women were less likely than nulliparous women to receive intrapartum oxytocin [24].

We also found a correlation between the mean cumulative dose of oxytocin administrated during labour and the use of epidural anaesthesia. Other studies have reported similar findings, both in terms of more frequent use of oxytocin administration after the epidural anaesthesia commenced [24, 25] and in the more frequent use of epidural anaesthesia following oxytocin augmentation of labour [26, 27].. This may be explained, in part, by the more painful nature of oxytocin-stimulated contractions and restrictions in movement that many women face when continuous electronic foetal monitoring is required during labour induction or augmentation [28].

Although some studies have reported negative associations between intrapartum oxytocin administration and neonatal outcomes [24, 29], we did not find any correlation between the mean cumulative dose of oxytocin administrated and Apgar scores and neonatal weight. However, using a more sensitive indicator of neonatal wellbeing, such as pH values in neonates, may lead to different results. A recent study showed the possible long-term effect of dose-dependent exposure to oxytocin [30]. An increased risk of autism spectrum disorders was noted in male who were exposed to long-term exposure and a large, cumulative dose of synthetic oxytocin during childbirth [30].

In our study, 84% of women birthed vaginally following labour augmentation, similar to the proportion of 87% found in a Danish study [29] but considerable higher than the 51% reported in Bugg et al.’s (2005) study with nulliparous women [31].

We did not find any correlation between the mean cumulative dose of oxytocin administrated to women and the mode of delivery. Similar findings were reported in studies comparing the high, and low-dose augmentation, where the higher doses of oxytocin administrated did not lower the risk of CS [22]. A systematic review concluded that early administration of oxytocin did not affect the mode of birth [8]. However, some studies have demonstrated a correlation between oxytocin administration and mode of delivery, including a lower risk of CS when compared to expectant management [27] but overall higher risk of intrapartum interventions [24, 26, 31]. Systematic reviews show that high-dose inductions and high-dose augmentation did not affect caesarean section rates [7, 32].. We were unable to find any other studies reporting on the correlation between the cumulative dose of oxytocin administered and mode of birth.

Ours findings revealed that most hospitals in Warsaw did not have written protocols on intrapartum oxytocin administration for induction or augmentation of labour. Even in units where protocols were available and compliant with the national guidelines, the actual administration of oxytocin did not follow the recommended regimen in 94% of observed labours.

While one study conducted in Germany found that 69% of participating units had protocols on oxytocin administration [3], they did not explore actual adherence to the protocol. The study by Jackson showed that establishing a collaboration between various care providers to build a consensus-driven, evidence-based approach to the use of oxytocin resulted in a significant increase (from 73 to 100%) of adherence of practice to the guidelines [6]. Studies showed that the use of oxytocin in accordance with guidelines was associated with several significant clinical outcomes, including a decrease in caesarean births for foetal distress based on electronic foetal monitoring, decreases in the length of the first stage of labour, and the decrease in the maximum dose of oxytocin [33].

With few exceptions, the oxytocin infusion was continued in all the observed labours, despite the national guidelines, [5] and information in Summary of Product Characteristics for oxytocin, [34] stating that the infusion may be discontinued when labour becomes established, i.e., when cervical dilatation reaches 5 cm. In the light of the latest reports that show the benefit of discontinuing the infusion once established labour is achieved [35, 36], observed practices require further analysis. A double blind randomized controlled trial confirmed that routine discontinuation of oxytocin stimulation may lead to a small increase in the caesarean section rate but significantly reduced the risk of uterine hyperstimulation and abnormal fetal heart rate patterns [37]. Since too few women in our test group had oxytocin discontinued after reaching active labour, we could not make similar comparisons between the groups in our study.

The guidelines for the supply of oxytocin proposed by PSOGO do not apply to particular situations such as twin pregnancies, pregnancies with previous caesarean section, pregnancies in obese women. They also lack information on the dose of oxytocin that should be set after discontinuation of infusion for the time epidural anaesthesia is provided.

Even assuming that there were situations in our study group that required non-standard behaviour, it seems unlikely that this would apply to over 90% of those surveyed.

Jackson et al. showed that the lack of adherence to oxytocin protocols resulted primarily from the insufficient consensus between care providers and the relevant literature regarding specific, restrictive dosing of oxytocin. Cooperation in building the standards and reaching the consensus may significantly improve the adherence of practice with guidelines [38].. These findings are particularly significant for Poland, where national guidelines for oxytocin administration were issued by obstetricians only.

Our study showed that deviations from the protocol for labour augmentation were associated with increasing time interval to dose escalation. This may be due to the burden being placed on staff when caring for several labouring women at the same time and the inability to increase the dose at an exact interval. Investigating the cause of this condition, requires more investigation.

The Jackson study found that increasing compliance was associated with a statistically significant reduction in the dose of oxytocin used during labour and a reduction in the time between initiating oxytocin administration and the birth of the child [6].

The two maternity units in this study, despite having similar protocols on oxytocin administration, differed considerably in the rates of labour induction and augmentation, as well as in the rates of vaginal births after labour induction. Similar discrepancies among units were reported in studies from Sweden and the UK, suggesting that care offered in different units is based on clinicians’ attitudes and preferences rather than on scientific research [24, 27]. As stated previously, the oxytocin administration regimens were not followed in 510 (93.6%) of the labours observed during our study. Although there is no scientific consensus on the optimal oxytocin administration dose or regimen, clinicians should follow their unit’s guidelines unless exceptional circumstances are present.

## Limitations

One of the limitations of our study was the inability to access and analyse data from all women undergoing induction or augmentation of labour in the two units. Analysis of data from all women may have resulted in different outcomes.

We conducted the study in tertiary-level urban units providing specialist care to women who may have had complicated medical and obstetric history. Recruitment bias may have affected the rates of therapeutic interventions, including induction and augmentation rates, and it would be beneficial to conduct a similar study in units catering mostly for healthy women.

## Conclusions

We did not observe any effect of the mean cumulative oxytocin dose on short-term perinatal outcomes. Further research exploring the impact of oxytocin on long-term outcomes would be beneficial. In most observed labours, the practice of oxytocin administration was non-compliant with national guidelines. A further research on the reasons for the continuation of oxytocin infusion after achieving established labour is needed.

In countries with low compliance of practices with standards, such as Poland, measures should be taken to investigate the reasons for non-compliance, and efforts should be put in place to support practice adherence to guidelines. As indicated by previous research, cooperation between different professional groups of care providers in building guidelines, proved to be very effective in improving compliance of practice with those guidelines [6]. Thus, in case of Poland, cooperation between midwives and obstetricians, as two major professional groups of maternity care providers, should be considered in building nationwide standards of maternity care.

## Supplementary Information

**Additional file 1.** Observation chart for recording oxytocin administration

**Additional file 2.** Supplementary Table 1.

## Data Availability

The datasets used and/or analysed during the current study are available from the corresponding author on reasonable request.

## Abbreviations

BMI: Body mass index
CS: Caesarean section
mU: MilliUnits
IU: International Units
mU/min: MilliUnits per minute
ml: Mililiter
OECD: the Organization for Economic Co-operation and Development
PSOGO: The Polish Society of Gynaecologists and Obstetricians
SD: Standard deviation
