# Supplementary material for: Oxytocin administration for induction and augmentation of labour in polish maternity units – an observational study
Source: BMC Pregnancy Childbirth. 2021 Nov 11;21:764. doi: 10.1186/s12884-021-04190-w (PMC8582102; doi:10.1186/s12884-021-04190-w)
Supplement: Supplementary file 1 — Additional file 1. Observation chart for recording oxytocin administration [file 12884_2021_4190_MOESM1_ESM.docx]

ID…………. age:………. No. obstetric records/medical records: ………….. No. of previous pregnancies: ………… No. of previous births: Gestational age: …………

Oxytocin was administrated earlier yes no

Singleton pregnancy twin pregnancy previous caesarean section epidural, start at: ……………..

- Induction with oxytocin, indications: ……………………………………………………...
- Augmentation with oxytocin, indications: …………………………………………………......................

Mode of birth Spontaneous vaginal (date and time) …………………………. forceps Ventouse

CS (date and time) …………………………. indications: ………………………….

| Exact time of escalation |  |  |  |  |  |  |  |  |  |  |  |  |  |  |  |  |  |  |  |  |  |  |  |  |  |  |  |  |
| --- | --- | --- | --- | --- | --- | --- | --- | --- | --- | --- | --- | --- | --- | --- | --- | --- | --- | --- | --- | --- | --- | --- | --- | --- | --- | --- | --- | --- |
| Dosage |  |  |  |  |  |  |  |  |  |  |  |  |  |  |  |  |  |  |  |  |  |  |  |  |  |  |  |  |
| Bolus |  |  |  |  |  |  |  |  |  |  |  |  |  |  |  |  |  |  |  |  |  |  |  |  |  |  |  |  |
| Cervix dilatation |  |  |  |  |  |  |  |  |  |  |  |  |  |  |  |  |  |  |  |  |  |  |  |  |  |  |  |  |

Volume of the solution fluid/no. of oxytocin units: ……… ml /……. mU type of infusion fluid: 0.9% NaCl other: …………..

Prior to birth (administrated): ……… ml infusion After birth (continuation): ………….ml infusion

Additionally administrated ..................... amp in III stage of labour, ……………amp in IV stage of labour, Pabal

Volume of infusion left after stopping …… ml blood loss ………………ml other drugs preventing extensive bleeding

Neonate (Apgar) after 1 min:……… after 5 min:………after 10 min:………

Name of a person who filled in the chart: …………………………………..
