# Supplementary material for: Oxytocin administration for induction and augmentation of labour in polish maternity units – an observational study
Source: BMC Pregnancy Childbirth. 2021 Nov 11;21:764. doi: 10.1186/s12884-021-04190-w (PMC8582102; doi:10.1186/s12884-021-04190-w)
Supplement: Supplementary file 2 — Additional file 2. Supplementary Table 1. [file 12884_2021_4190_MOESM2_ESM.docx]

|  | Unit A (N=1527) | % | Unit B (N=518) | % | Total  (N=2045) | % | *p-value* |
| --- | --- | --- | --- | --- | --- | --- | --- |
| **Labours without oxytocin** | **1065** | **69,74** | **233** | **44,98** | **1298** | **63,47** | *x2(1*)=102,31 **p=0,0000** |
| **Labours with oxytocin** | **462** | **30,24** | **285** | **55,02** | **747** | **36,53** |  |
| Labours after oxytocin augmentation | 214 | 14.01 | 138 | 26.64 | 352 | 17.21 | *x2(1)=,*31  p=0,5764 |
| Labours after oxytocin induction | 248 | 16.23 | 147 | 28.38 | 395 | 19.32 |  |

Suplemental Table 1 Labours without oxytocin and with oxytocin (N=2045).
